# Obituary: Erney Felicio Plessmann de Camargo

**DOI:** 10.31744/einstein_journal/2023ED0000

**Published:** 2023-03-10

**Authors:** Luiz Vicente Rizzo

**Affiliations:** 1 Instituto Israelita de Ensino e Pesquisa Albert Einstein Hospital Israelita Albert Einstein São Paulo SP Brazil Instituto Israelita de Ensino e Pesquisa Albert Einstein, Hospital Israelita Albert Einstein, São Paulo, SP, Brazil.

**Figure:**
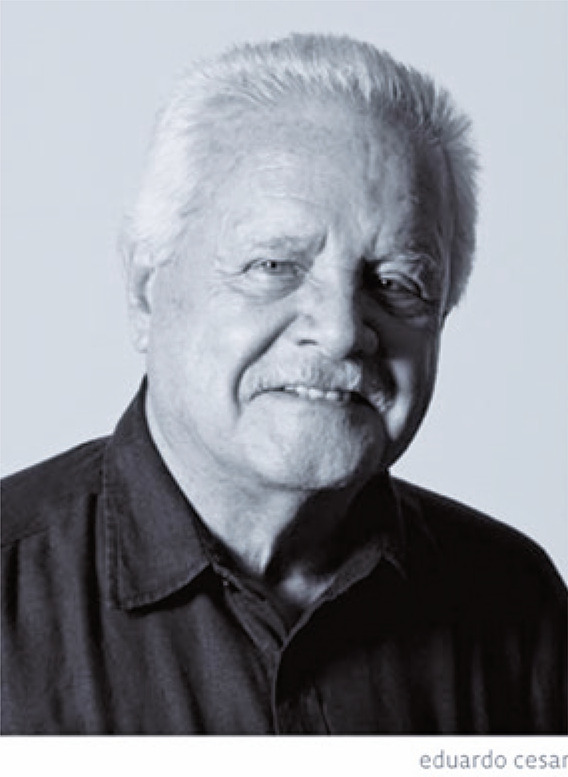
Source: Pesquisa FAPESP.

Erney Felicio Plessmann de Camargo, Professor Emeritus at the *Universidade de* *São Paulo* , a member of the Brazilian Academy of Sciences and honorary member of the National Academy of Medicine has passed. When Professor Erney took over *Conselho Nacional de Desenvolvimento Científico e Tecnológico* he revolutionized the agency, instituting many procedures that were fundamental to move Brazilian science forward. Despite the lack of appropriate funding, he managed to oversee an outstanding growth of scientific productivity in Brazil. Erney was a friend and a mentor. His role at the Scientific Advisory board of the Institute since 2010 was instrumental to the scientific success we achieved. He used to say that “science is done by scientists and that is what they should do” that thought helped to shape the idea of the Researcher Support Office at the *Hospital Israelita Albert Einstein* , which provides all the pre-award and post-award help to scientists at Einstein amongst other support services for the advancement of science. Erney will be sorely missed. We will try our best to keep alive his vision of science and innovation as the way for mankind to overcome the ills that plague us. With our most felt condolences to his family and friends we bid goodbye to one of the great scientists and research administrators that our nation has ever produced.

